# A Choosing Wisely top-5 list to support general practitioners in Austria

**DOI:** 10.1007/s10354-021-00846-6

**Published:** 2021-05-10

**Authors:** Anna Glechner, Susanne Rabady, Herbert Bachler, Christoph Dachs, Maria Flamm, Reinhold Glehr, Kathryn Hoffmann, Renate Hoffmann-Dorninger, Gustav Kamenski, Matthias Lutz, Stephanie Poggenburg, Wilfried Tschiggerl, Karl Horvath

**Affiliations:** 1 Department for Evidence-based Medicine and Clinical Epidemiology, Cochrane Austria, Danube University Krems, Dr.-Karl-Dorrek Straße 30, 3500 Krems a.d. Donau, Austria; 2 Department of General Medicine and Family Practice, Karl Landsteiner University of Health Sciences, Krems, Austria; 3 General Medicine and Family Medicine, Medical University Innsbruck, Innsbruck, Austria; 4 Institute of General Practice, Family Medicine and Preventive Medicine, Paracelsus Medical University, Salzburg, Austria; 5 Institute of General Practice and Evidence-Based Health Research, Medical University Graz, Graz, Austria; 6 Department for General Medicine and Family Practice, Center for Public Health, Medical University of Vienna, Vienna, Austria; 7 Karl Landsteiner Institute for Systematics in General Medicine, Angern, Austria; 8 Austrian Institute for General Medicine, Klagenfurt, Austria

**Keywords:** Overuse, Overdiagnosis, Family medicine, Antibiotics, Low back pain, Überbeanspruchung, Überdiagnose, Familienmedizin, Antibiotika, Schmerzen im unteren Rücken

## Abstract

From a pool of 147 reliable recommendations, ten experts from the Austrian Society of General Practice and Family Medicine selected 21 relevant recommendations as the basis for the Delphi process. In two Delphi rounds, eleven experts established a top‑5 list of recommendations designed for Austrian family practice to reduce medical overuse. Three of the chosen recommendations address the issue of antibiotic usage in patients with viral upper respiratory tract infections, in children with mild otitis media, and in patients with asymptomatic bacteriuria. The other two “do not do” recommendations concern imaging studies for nonspecific low back pain and routine screening to detect prostate cancer. A subsequent survey identified the reasons for selecting these top‑5 recommendations: the frequency of the issue, potential harms, costs, and patients’ expectations. Experts hope the campaign will save time in educating patients and provide legal protection for omitting measures.

## Abstract (de)

Aus einem Pool von 147 verlässlichen Empfehlungen wählten 10 Experten der Österreichischen Gesellschaft für Allgemein- und Familienmedizin 21 relevante Empfehlungen als Grundlage für den Delphi-Prozess aus. In 2 Delphi-Runden erstellten 11 Experten eine Top-5-Liste von Empfehlungen, um eine medizinische Überversorgung in österreichischen Hausarztpraxen zu reduzieren. Davon befassen sich 3 mit der Frage des Antibiotikaeinsatzes bei viralen Infekten der oberen Atemwege, bei leichter Otitis media von Kindern und bei asymptomatischer Bakteriurie. Die beiden anderen „Don’t-do-Empfehlungen“ betreffen bildgebende Untersuchungen bei unspezifischen Rückenschmerzen sowie Routineuntersuchungen zur Früherkennung von Prostatakrebs. In einer anschließenden Umfrage wurde ermittelt, welche Gründe den Ausschlag für die Auswahl dieser Top-5-Empfehlungen gaben: die Häufigkeit des Themas, mögliche Schäden, Kosten und die Erwartungen der Patienten. Die Experten erhoffen sich von der Kampagne eine Zeitersparnis bei der Patientenaufklärung und eine rechtliche Absicherung bei unterlassenen Maßnahmen.

## Introduction

Medical overuse, defined as “unnecessary tests and treatments that lack patient benefit or bear the potential to cause harm,” has become a major concern in highly industrialized countries and may affect 42% of patients’ treatment plans [1]. Patient pressure as perceived by doctors has been shown to encourage drug prescribing and test requisitions against the physician’s better judgment [2, 3]. Another relevant trigger for overuse has been attributed to defensive medicine: in cases of diagnostic uncertainty, taking action is usually considered safer than doing nothing [4].

A worldwide campaign against overuse, called “Choosing Wisely,” has been launched in 24 countries around the globe [5]. The Choosing Wisely campaign, initiated by the American Board of Internal Medicine, was designed as a support tool to educate the public and facilitate doctor–patient and professional communication [6]. For diagnostic tests or treatments that do not prove beneficial for most patients for whom they are commonly prescribed, medical specialty societies create their own top lists.

In Austria, a high utilization of health care services exists due to the restriction-free access to all health insurance services without any associated costs for patients [7]. Data from 2013 from the Lower Austrian Health Insurance Company revealed that 34 low-value services were provided to more than 240,000 beneficiaries [8]. In 2017, the Austrian Choosing Wisely initiative, *Gemeinsam gut entscheiden*, was founded with the aim of counteracting medical overuse [9]. So far, five Austrian medical societies have developed top lists: geriatrics and gerontology, general practice and family medicine, public health, gynecology and obstetrics, and nephrology. The aim of our work is to present the five recommendations that were judged most relevant by the Austrian Society of General Practice and Family Medicine regarding overuse in the field of primary care and which are based on robust enough evidence to justify being promoted among laypeople as well as professionals. We also analyzed the criteria used in the selection process.

## Methods

The methods for our project were a combination of literature search, Delphi process, and questionnaire survey.

### Literature search

All published recommendations of the US Choosing Wisely initiative were identified through the website of the American Board of Internal Medicine Foundation [6]. Additionally, a search for recommendations from mid-European Choosing Wisely initiatives through the websites of the Diana Health project of the *Centro de Investigación Biomédica en Red de Epidemiología y Salud Pública* [10] and the Less is More project was performed [11]. The literature searches were performed in April 2017 and updated in October 2019. Recommendations were judged to be trustworthy if they had equivalent recommendations in German guidelines ranked class S3, i.e., the highest level, or if the development process was judged to be of high methodological quality and meta-literature supporting the recommendation was cited [12].

### The Delphi process

The Delphi process is a systematic, multi-stage survey procedure developed by the American RAND Corporation in 1963 and is often used in varied form to assess whether there is consensus on a topic [13, 14]. The survey is carried out anonymously to minimize any influence of dominant group members.

Within the Delphi process, the five most important recommendations for the field of family medicine were rated on a Likert scale from 1 (less important) to 5 (very important) [15]. We calculated a mean and associated standard deviation for each of the recommendations evaluated. From the second round onwards, the experts re-evaluated all of the recommendations, whereby they were presented with the overall result of the previous round and their own previous assessment. If evaluations had varied greatly, we would have used an additional discussion and a further evaluation until a consensus was reached; however, that proved unnecessary. Recommendations whose ratings achieved a mean score of at least 4.0 and whose standard deviations did not exceed 1.0 were included in the final top‑5 list.

### Selection of experts for the Delphi process

The experts invited to take part in the selection process were chosen according to their expertise in the field of general practice. We identified those members of the Austrian society of General Medicine who were experienced in both daily practice and researching or teaching in the academic field of general medicine, and invited them to take part in the study. Eleven of the 16 colleagues who were invited agreed to participate in the process. Each of them signed a conflict of interest declaration form.

### Questionnaire on reasons for selection of recommendations

A questionnaire was developed to determine the motivations and triggers that led the experts to select their recommendations for the top‑5 list. The first part of the questionnaire was designed to disclose the reasons behind the selection. It contained the core requirements for recommendations as formulated by the Choosing Wisely initiative, i.e., frequency of the problem and potential harm [6], as well as external influences [16] on shared medical decision-making. The second part of our survey ascertained whether the raters anticipated that an Austrian Choosing Wisely campaign could reduce the amount of time spent on educating patients as well as reduce external pressure experienced by physicians, and if the campaign could alleviate fear of litigation as one factor leading to defensive behavior [4].

## Results

### Selection of recommendations

**Fig. 1:**
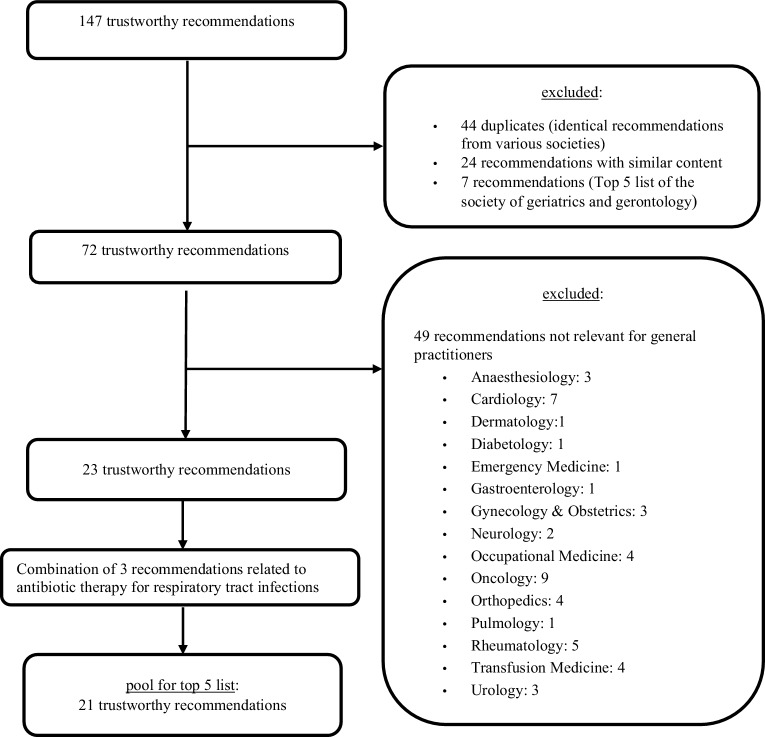
Preselection of trustworthy recommendations for inclusion in the Delphi process

Our search on websites of international Choosing Wisely initiatives resulted in 147 reliable recommendations. We excluded 44 duplicates (identical recommendations from various medical societies), 24 recommendations with similar content, and seven recommendations that were already covered by the top‑5 list of the Society of Geriatrics and Gerontology [17]. From the remaining pool of 72 recommendations, a team of ten experts from the Austrian Society of General Practice and Family Medicine selected those that were most relevant for general practitioners. A total of 49 recommendations were excluded from the pool due to their relevance for medical specialities other than general practice (Fig. 1). Finally, a pool of 23 recommendations was available for further assessment and for the selection of the top 5. Three of the recommendations related to antibiotic therapy for respiratory tract infections and were therefore combined, so that 21 recommendations were available for the final Delphi survey. Fig. 1 illustrates the selection of recommendations for the pool of the top‑5 list. The top‑5 list was created in a two-step Delphi process, which took place from July 2018 to September 2018.

#### First Delphi round

Eleven experts assessed the pool of 21 recommendations on a Likert scale from 1 (least important) to 5 (most important). *Not performing imaging studies for nonspecific low back pain* achieved the highest mean value among the eleven experts (4.5 ± 1.0), with eight of eleven rating it most important. Three recommendations contained the advice to avoid antibiotics for different indications (Table 1). *Avoid prescribing antibiotics for upper respiratory tract infections *was rated a 4 or a 5 (very or most important) by eight of the eleven experts. The other two indications for which antibiotics should not be used routinely were *otitis media in children aged 2 to 12 years* and *asymptomatic bacteriuria*. The fifth recommendation was indirectly linked to the unnecessary prescription of antibiotics and was about *not obtaining a urine culture if there are no symptoms indicating a urinary tract infection*. There was a broad consensus within the team on the importance of the first five recommendations with mean scores above 4.0 (4.1–4.5; standard deviation, SD: 0.7–1.2). The recommendation *to avoid antibiotics for upper respiratory tract infections* achieved a mean score of 4.2; however, the standard deviation was 1.2 as one expert rated this item as a 1.

**Table 1:** Results of Delphi rounds 1 and 2, rated by 11 assessors

Recommendation	Ratings Likert scale^a^					Mean value (SD)
	1	2	3	4	5	
*Delphi round 1*						
Don’t do imaging for low back pain within the first 6 weeks, unless red flags are present	0	1	1	1	8	4.5 (1.0)
Don’t prescribe antibiotics for otitis media in children aged 2–12 years with non-severe symptoms where the observation option is reasonable	0	1	1	3	6	4.3 (1.0)
Avoid prescribing antibiotics for upper respiratory infections	1	0	2	1	7	4.2 (1.2)
Don’t treat asymptomatic bacteriuria with antibiotics	0	1	1	5	4	4.1 (0.9)
Don’t obtain a urine culture unless there are clear signs and symptoms that localize to the urinary tract	0	0	2	6	3	4.1 (0.7)
*Delphi round 2*						
Avoid prescribing antibiotics for upper respiratory infections	0	0	0	4	7	4.6 (0.5)
Don’t do imaging for low back pain within the first 6 weeks, unless red flags are present	0	0	2	2	7	4.5 (0.8)
Don’t prescribe antibiotics for otitis media in children aged 2–12 years with non-severe symptoms where the observation option is reasonable	0	0	1	4	6	4.5 (0.7)
Don’t treat asymptomatic bacteriuria with antibiotics	0	1	1	3	6	4.3 (1.0)
Don’t routinely screen for prostate cancer using a prostate-specific antigen (PSA) test or digital rectal exam	0	1	1	5	4	4.1 (0.9)

*SD* standard deviation

^a^Likert scale: 1 = least important, 2 = less important, 3 = important, 4 = very important, 5 = most important

#### Second Delphi round

In order to establish a consensus regarding the top‑5 recommendations, a second survey was necessary. All the experts from the first Delphi round reevaluated the 21 recommendations. The four first-ranked recommendations were each identified twice (in the first and second Delphi round) by a majority as the four most important recommendations with mean scores of 4.3–4.6 (standard deviations 0.5–1.0). The fifth recommendation varied in the two rounds. In the second round, the experts chose antihypertensive treatment in older persons as the fifth recommendation. Since this recommendation was subject to international discussions, the team agreed that the sixth recommendation concerning routine PSA screening should take precedence, which was rated as 4 or 5 by nine of eleven experts (mean score: 4.1; SD: 0.9).

### Survey: reasons for the selection of recommendations and expectations for the campaign

Ten experts were asked to rate five blocks of selection criteria for each of the five recommendations according to their importance on a scale of 1 (very good reason) to 5 (no reason at all). Two questions about the expectations of the campaign were agreed upon. The survey was performed from October to November 2019 among all experts involved in the Delphi process, excluding R. S., one of first authors of this study.

Table 2 illustrates the results. In the following section we present the mean values and the corresponding standard deviations.

**Table 2:** Reasons to rate the top‑5 list recommendations, factors that contribute to overuse, and expectations regarding the Choosing Wisely campaign

Argument		Avoid prescribing antibiotics for URTI	Don’t do imaging for LBP within the first 6 weeks, unless red flags are present	Don’t prescribe antibiotics for otitis media in children aged 2–12 years with non-severe symptoms	Don’t treat asymptomatic bacteriuria with antibiotics	Do not perform routine screening to detect prostate cancer
Likert scale: 1 to 5; mean values (SD)						
Frequent topic in daily practice and therefore particularly important		1.4 (± 1.2)	1.5 (± 0.7)	1.5 (± 0.5)	1.7 (± 0.9)	1.9 (± 0.9)
Improper decisions have a special impact on	a) Patients’ health	1.6 (± 0.7)	2.5 (± 1.2)	2 (± 0.8)	1.4 (± 0.7)	1.8 (± 1.0)
	b) Costs	2.9 (± 1.0)	1.6 (± 0.7)	3.4 (± 0.7)	3.1 (± 0.3)	2.4 (± 0.8)
	c) Does not apply	5.0 (± 0.0)	4.3 (± 0.9)	4.8 (± 0.4)	4.2 (± 1.2)	4.8 (± 0.4)
Medical uncertainty among GPs causing procedural deviations		2.9 (± 0.8)	2.3 (± 1.0)	2.3 (± 0.9)	2.5 (± 0.7)	2.6 (± 0.7)
Pressure on physicians through	a) Patients	2.3 (± 1.3)	1.4 (± 0.7)	2.3 (± 1.0)	2.8 (± 1.3)	1.7 (± 0.6)
	b) Hospitals	3.4 (± 0.8)	2.3 (± 1.5)	3.8 (± 0.6)	3.1 (± 1.2)	2.5 (± 0.7)
	c) Specialists	3.1 (± 1.2)	2.1 (± 1.6)^a^	3.1 (± 0.8)	3 (± 0.8)	1.7 (± 0.9)
	d) GP colleagues	4.1 (± 0.6)	4.0 (± 0.7)	3.0 (± 1.1)	4.1 (± 0.6)	3.1 (± 0.8)
	e) Patients’ relatives	2.8 (± 1.0)	2.7 (± 1.3)	1.9 (± 0.9)	3.1 (± 1.2)	2.8 (± 1.2)
	f) Insurance companies	4.3 (± 0.7)	4.0 (± 0.8)	4.2 (± 0.9)	3.8 (± 1.2)	3.5 (± 0.9)
	g) Other healthcare professions	4.0 (± 1.1)^b,c^	3.6 (± 1.6)^b^	3.9 (± 0.7)	2.9 (± 1.2)^c,d^	3.9 (± 0.8)
	h) Others (please specify)	4.1 (± 1.1)	4.6 (± 0.7)	4.1 (± 0.6)	4.4 (± 0.5)	4.3 (± 0.7)
	i) Does not apply	3.8 (± 1.5)	4.5 (± 0.9)	4.8 (± 0.4)	4.0 (± 1.6)	4.8 (± 0.4)
The top‑5 recommendations will provide legal protection		2.1 (± 1.1)	1.9 (± 1.2)	1.8 (± 0.8)	2.2 (± 0.6)	1.9 (± 0.8)
I expect the Choosing Wisely campaign to help	a) Reduction of time expenditure	8/10	7/10	5/9	4/8	8/9
	b) Reduction of external pressure on decision-making	6/10	6/10	9/9	6/8	8/9
	c) Other (please specify)	1/10^e^	1/10^f^	0	1/8	0
	d) None applies	0	0	0	1/8	0

Likert scale: 1 = very good reason, strongly agree; 2 = good reason, agree; 3 = undecided, neutral; 4 = no good reason, disagree; 5 = no reason at all, strongly disagree

*LBP* low back pain, *URTI* upper respiratory tract infections, *GP *general practitioner

^a^Four of the experts mentioned pressure from physiotherapists

^b^One expert mentioned pressure from nursing staff

^c^One expert mentioned pressure from pharmacists

^d^Two experts mentioned pressure from nursing staff, especially in nursing homes

^e^Reduction of antibiotics prescription

^f^Reduction of unnecessary imaging

#### Frequent topic in daily practice

The argument* Frequent topic in daily practice and therefore particularly important* was considered relevant for the selection process by all ten experts for all recommendations in the top‑5 list. This concern yielded the highest overall agreement, ranging from a weighted average of 1.4 (± 1.2) for *Avoid prescribing antibiotics for upper respiratory tract infections*, which had achieved the highest mean value in the Delphi process, to 1.9 (± 0.9) for the recommendation *not to perform routine screening to detect prostate cancer*.

#### Potential harms

The criterion *Improper decisions have a special impact on patients’ health* met with the second highest overall approval (range: 1.4 ± 0.7 to 2.5 ± 1.2). For all three recommendations concerning unnecessary use of antibiotics, experts judged possible harm as a good or very good reason for the selection. Potentially negative impact on health was considered a major concern by eight of ten experts (mean value: 1.8 ± 1.0) for routine screening for prostate cancer.

#### Unnecessary costs

The argument* Improper decisions have a special impact on costs* was altogether attributed less importance in choosing the recommendations for the top‑5 list. The highest impact of costs was seen for the recommendation concerning *imaging studies for non-specific low back pain*, with nine experts out of ten agreeing (mean value: 1.6 ± 0.7).

#### Medical uncertainties

Three of the recommendations on the top‑5 list were also chosen by experts because *medical uncertainties* could be a reason for non-adherence to the guidelines. Seventy percent (7 of 10) suspect that family doctors were unsure (rating 1 or 2) about whether or when to recommend* imaging studies for non-specific low back pain *(mean value: 2.3 ± 1.0). Sixty percent (6 of 10) also stated that *uncertainty* could lead to more antibiotics being prescribed unnecessarily for children with otitis media (mean value: 2.3 ± 0.9) or for asymptomatic bacteriuria (mean value: 2.5 ± 0.7).

#### Pressure on doctors through requests from other parties

The majority of our experts (8 of 9) agreed or strongly agreed that patient pressure has a role in driving decisions against the recommendation *not to perform imaging studies for nonspecific low back pain* (mean 1.4 ± 0.7). Consistently, nine out of ten experts agreed that patients often expect physicians *to routinely screen for prostate cancer using PSA testing* (mean value: 1.7 ± 0.6). Also, the prescription of antibiotics for upper respiratory tract infections or otitis media in children is often demanded by patients or parents, which may contribute to overuse (Table 2).

Pressure from specialists influenced decisions related to *performing imaging studies for non-specific low back pain* (mean value: 2.1 ± 1.6) against recommendations. Seven out of nine experts agreed or strongly agreed with this, while two experts disagreed. Four of the experts explicitly mentioned pressure from physiotherapists. Nine of ten voters agreed or strongly agreed that specialists enhance decisions to deviate from the recommendation *not to routinely screen for prostate cancer* (mean value: 1.7 ± 0.9).

#### Reducing the time required for education

The greatest agreement of the potential of the Choosing Wisely campaign to save time in educating patients was achieved by the recommendation *not to perform routine screening for prostate *cancer (8 of 9). Eighty percent of the experts thought that the campaign would save time when explaining why antibiotics should not be prescribed for upper respiratory tract infections (the recommendation ranked first in the Delphi process). Seventy percent expected this for the second-ranked recommendation *no imaging should be performed for non-specific low back pain* (7 of 10) and 56% (5 of 9) for the recommendation *not to administer antibiotics for children with non-severe otitis media.*

#### Reduction of external pressure

Nine voters expected reduced pressure on decision-making processes as a result of the campaign for the recommendation *not to prescribe antibiotics for non-severe otitis media in children above 2 years, *and eight of those nine experts expected less pressure with respect to the recommendation *not to routinely perform PSA screening*.

#### Legal support

Legal support was an issue for all the selected recommendations (Table 2). For example, eight of the ten experts agreed or strongly agreed that the Choosing Wisely campaign will provide legal protection concerning two of the recommendations regarding antibiotics: *not to prescribe antibiotics for upper respiratory tract infections* (mean value: 2.1 ± 1.1) and for* otitis media in children aged 2–12 years with non-severe symptoms *(mean value: 1.8 ± 0.8).

## Discussion

Three of the recommendations on the top‑5 list relate to the use of antibiotics. Those recommendations are (1) *to only use antibiotics when indicated in patients with respiratory tract infections,* (2) *to choose the “watch and wait” option before prescribing them in children with otitis media,* and (3) *to avoid prescribing them in asymptomatic bacteriuria.* About 80 to 90% of all oral antibiotics are prescribed in primary care, half of them for respiratory infections and one-sixth for urinary tract infections [18]. The reasons for prescribing antibiotics as identified by research are complex and involve patient expectations, diagnostic insecurity, and limited time resources [19, 20]. Most experts (6 of 9) agreed that pressure from patients was an important reason to select the recommendation *to avoid antibiotics for upper respiratory tract infections* for the top‑5 list, as most patients believe that antibiotics are effective for treating viral infections [21]. Patients frequently expect to be spared another consultation when receiving antibiotics on the spot and the concept of antibiotic resistance is difficult to understand [22]. Significantly, according to doctors’ perceptions, pressure to use antibiotics for mild otitis media in children is often exerted by parents [23]. In addition, our experts assessed medical uncertainty as a reason for overprescribing in the treatment of children with non-severe otitis media.

Concerning the recommendation *not to perform imaging studies for non-specific low back pain, *seven of ten experts suspect that family doctors are unsure (rating 1 or 2) about whether or when to recommend these studies. Many abnormalities are present in asymptomatic persons and may reflect normal signs of ageing [24], while patients with low back pain may have no marked disc degeneration changes on imaging [25]. Informing patients that imaging studies could reveal abnormalities leading to unintended harms might prove to be challenging [26]. Even among our experts, only five out of ten thought that harm brought about by overuse of imaging studies in low back pain is a reason to select the recommendation, which leads to the assumption that possible harm from overdiagnosis is not generally a prioritized consideration. Pressure for imaging is also experienced from specialists and weighs on physicians, since it can enhance fear of litigation. Accordingly, experts put hopes on the Choosing Wisely campaign to help relieve decision-making pressure, as well as to reduce fear of litigation. Regarding imaging, the cost factor was also considered an objective for the campaign. Costs were not considered a major issue regarding any of the other recommendations.

Another diagnostic procedure for which pressure from patients as well as specialists was considered to have significant impact was* not to perform routine screening to detect prostate cancer*. The level of information about the benefits and risks associated with a PSA test is often insufficient [27]. This lack of information may compromise a patient’s ability to make informed decisions about whether to perform a PSA test or not and may result in a strong request for testing. Patient requests and worries are factors that have a great influence on primary care physicians for ordering PSA tests in men without any clinical suspicion of prostate cancer [28]. These findings are clearly supported by our study. Our experts considered patients’ expectations to strongly influence decision-making, particularly related to diagnostic testing (imaging for nonspecific low back pain and PSA screening).

Our survey has several limitations. First, our Delphi survey was based on existing “Do not do recommendations” of international Choosing Wisely initiatives. Therefore, the top‑5 list may reflect the topics discussed in most of the other countries by general practitioners, but may miss items that are only relevant in Austria. For instance, in Austria the demarcation line between primary care and secondary care is less clearly defined, which may lead to competition between professions and could contribute to overuse [7]. Second, we did not assess the significance of limited time resources to implement the recommendations. Studies suggest that high-prescribing practices had a higher practice volume and were more often located in deprived or rural areas [29]. Nevertheless, our experts expect that the information campaigns can help save time with patient education and support more efficient time utilization in doctor–patient interactions.

The third limitation is the limited number of experts participating and the fact that they enlisted themselves following a call directed at all members of the Austrian Society of General Practice and Family Medicine. However, several experts of the team are involved in scientific research to improve general practice in Austria and all of them are experienced GPs as well as lecturers in this field. The results of our Delphi process therefore provide indications of common overuse issues that are relevant for Austria.

## Conclusion

The top‑5 list of the Austrian Society for General Practice and Family Medicine addresses overuse issues that are relevant for Austria. Three of the five recommendations address the use of antibiotics in Austrian primary care practices, while the remaining two concern diagnostic measures. Among the reasons to select the recommendations of the top‑5 list, the experts identified perception of external pressure by patients and specialists and fear of litigation as main the concerns to be addressed by the campaign. More research is necessary to identify motivators and barriers for the implementation of the top‑5 list, created by the Austrian Society of General Practice and Family Medicine, in a broader target group, which is essential for the success of the Choosing Wisely campaign.
